# Analytical Model of Induction Machines with Multiple Cage Faults Using the Winding Tensor Approach

**DOI:** 10.3390/s21155076

**Published:** 2021-07-27

**Authors:** Javier Martinez-Roman, Ruben Puche-Panadero, Angel Sapena-Bano, Carla Terron-Santiago, Jordi Burriel-Valencia, Manuel Pineda-Sanchez

**Affiliations:** Institute for Energy Engineering, Universitat Politècnica de València, Camino de Vera s/n, 46022 Valencia, Spain; jmroman@die.upv.es (J.M.-R.); rupucpa@die.upv.es (R.P.-P.); asapena@die.upv.es (A.S.-B.); cartersa@alumni.upv.es (C.T.-S.); jorburva@die.upv.es (J.B.-V.)

**Keywords:** inductance tensor, induction machines, fault diagnosis, winding asymmetries

## Abstract

Induction machines (IMs) are one of the main sources of mechanical power in many industrial processes, especially squirrel cage IMs (SCIMs), due to their robustness and reliability. Their sudden stoppage due to undetected faults may cause costly production breakdowns. One of the most frequent types of faults are cage faults (bar and end ring segment breakages), especially in motors that directly drive high-inertia loads (such as fans), in motors with frequent starts and stops, and in case of poorly manufactured cage windings. A continuous monitoring of IMs is needed to reduce this risk, integrated in plant-wide condition based maintenance (CBM) systems. Diverse diagnostic techniques have been proposed in the technical literature, either data-based, detecting fault-characteristic perturbations in the data collected from the IM, and model-based, observing the differences between the data collected from the actual IM and from its digital twin model. In both cases, fast and accurate IM models are needed to develop and optimize the fault diagnosis techniques. On the one hand, the finite elements approach can provide highly accurate models, but its computational cost and processing requirements are very high to be used in on-line fault diagnostic systems. On the other hand, analytical models can be much faster, but they can be very complex in case of highly asymmetrical machines, such as IMs with multiple cage faults. In this work, a new method is proposed for the analytical modelling of IMs with asymmetrical cage windings using a tensor based approach, which greatly reduces this complexity by applying routine tensor algebra to obtain the parameters of the faulty IM model from the healthy one. This winding tensor approach is explained theoretically and validated with the diagnosis of a commercial IM with multiple cage faults.

## 1. Introduction

The growing importance of electrical machines, and especially SCIMs [1,2], in industrial production lines, electricity generation, and electric mobility, has sparked a growing demand of condition based monitoring (CBMs) systems, which help maintain their operation and avoid costly breakdowns of machines and production lines due to the sudden appearance of undetected IMs faults. Among IM machines, cage IMs are considered to be the most rugged and reliable ones, due to the robustness of the cage assembly. Nevertheless, in motors that directly drive high-inertia loads (such as fans), in motors with frequent starts and stops, or in case of poorly manufactured cage windings [3,4], bars or end rings can have failures, due to high mechanical and thermal stresses of the rotor cage, especially during the start-up process of line fed IMs [5,6]. These faults must be detected as early and fast as possible, because they can produce heat damage to the rotor core, an increase of the current for a given load, and a reduction of the torque and efficiency [3,7].

Responsive CBMs must be able to operate on-line, in a non-invasive way, so that any fault can be detected in an incipient state and corrective maintenance measures can be applied before it becomes a catastrophic one. Although the cage fault is a slowly developing one, it is important to deploy fast and simple diagnostic techniques that can be applied on-site, without the need for transmitting a huge amount of machine data to higher-level processing centres, so saving valuable communications bandwidth resources. This requires fast and simple fault diagnostic techniques, which can be implemented in embedded field devices, such as digital signal processors (DSPs) or field-programmable arrays (FPGAs) [8]. For example, a growing trend in the condition monitoring of induction motors is the use of smart sensors, attached to the motor frame, such as the SIEMENS Simotics Connect 400 [9], or the ABB Ability Smart Sensor [10], which performs the diagnostic procedure locally, and transmits the diagnostic results to the Internet of Things (IoT). Other scenarios that benefits from fast diagnostic techniques are companies dedicated to diagnosis responsible for monitoring big sets of motors, which might need several analyses in the case of alarm, needing a quick response of the motor state to avoid unnecessary stops [11].

Among the different fault diagnostic techniques that can be deployed in CBM systems, the use of digital twins is attracting a rising interest: a digital model of the IM is built, and the model outputs (currents and voltages) are compared with the quantities measured at the machine terminals. Divergences between the predicted and measured values, as well as the increase with time of these differences, are clear indications of a possible fault. Recent developments in this field propose to integrate discrete component prognosis in model-based CBMs of hybrid systems, with a new event-triggering mechanisms using degradation model selection [12]. This methodology enables even the prognosis of intermittent faults in discrete components, as shown in [13]. Digital twins of an IM can be built using different approaches. The finite elements method (FEM) provides highly accurate IM models [14], but it demands huge computing resources in terms of speed and processing power, especially when simulating non-symmetrical, faulty IMs. This hampers its use in low-power embedded units. On the contrary, analytical models, based on a circuital approximation, can be very fast, but they lack the precision of FEM models. Nevertheless, from a diagnostic point of view, it suffices that the analytical models can reproduce accurately the effects of the faults in the IM currents or voltages, and they can do this at a much higher speed and lower cost that FEM models [15]. As [16] points out, these analytical models allow finding the most important effects of cage asymmetry and require only the basic motor parameters. Another diagnostic area in which IM models are used is in the training of neural networks or expert systems for fault diagnosis, which need thousands of tests performed under different working conditions with controlled degrees of IM faults. In this area, again, the speed of analytical models can give them a decisive edge over FEM.

One of the main difficulties in the development of circuital models of the IM is the dependency of the mutual and self inductances of the windings, and their derivatives, on the rotor position. This is a complex, non-linear function, which depends on the windings configurations, and on their relative positions [15]. Besides, these configurations may become asymmetrical in case of cage faults, rendering useless many labor-saving procedures that can only be applied to symmetrical windings. Indeed, mutual and self inductances of rotor and stator windings must be recalculated for each type of fault. A common simplification is to consider only pure sinusoidal air-gap spatial waves, that is, approximating the winding inductances by their fundamental harmonic component. Nevertheless, the complex interactions between spatial and time harmonics that generate the characteristic fault harmonics in the machine current cannot be accurately reproduced by these simplified models, what prevents their use for fault simulation and diagnosis of SCIMs.

Diverse approaches have been presented in the technical literature for an accurate computation of the inductance matrix needed in analytical models of the SCIM under fault conditions. In [17,18], this matrix is obtained by direct measurements, in [19] a FEM model has been used for inductance computation, and in [20,21] a hybrid FEM-analytical method has been presented. In [22], a reduced-order model of the rotor cage is used to take into account non-sinusoidal magnetomotive (MMF) forces. Saturation and non-linearities of the magnetic circuit have been taken into account in circuital IM models using modified air gap length functions [23], the co-energy based method [15], or a complex multi-harmonic model [24]. In [25], the partial-inductance method has been proposed for obtaining the inductance matrix using an analytical solution of the air gap magnetic vector potential. Linear models allow for a further simplification, using a one-dimensional analysis in which the radial component of the flux density is determined as a function of the angular position of the coils along the air gap circumference [15]. Many formulas for determining the self and mutual inductances of an arbitrary pair of coils situated in the air gap zone have been presented in the technical literature, as in [26,27], and they are the base of the winding function approach (WFA) [23,28]. Nevertheless, this approach requires complex winding functions that depend on the relative position of the coils, on the coils MMF functions, on the permeance function of the air gap, and on the rotor position, leading to triple integrals for each pair of coils [15]. On the contrary, the winding tensor function approach [29] uses the conductor as the most basic unit, instead of the coil, and gives the self and mutual inductances of the IM windings applying routine tensor algebra functions, following Kron’s method [26,30].

The methodology proposed in this work greatly simplifies the process for calculating the parameters of the SCIM model under different rotor asymmetry conditions, using a novel approach: instead of obtaining directly the parameters of the SCIM under faulty conditions, which is a difficult computation that must be done for each type of fault or combination of faults, only the parameters of the healthy SCIM are obtained, using the winding tensor approach [29]. The parameters of the SCIM under any type of rotor asymmetry (bar breakages, end-ring breakages) are then obtained using simple connection tensors, whose elements are only zeros, ones or minus ones, and applying routine tensor algebra operations. It is proven, both theoretically and experimentally, that this simple approach is able to account for any type and number of rotor asymmetry faults, so avoiding a cumbersome setup of the equations of all the possible asymmetrical rotor circuits that correspond to these faults.

The paper’s structure is the following one. In Section 2, the analytical model of the SCIM is presented. In Section 3, the parameters of the SCIM are obtained in a primitive reference frame, where they have its simplest expression. In Section 4, the model of a SCIM in healthy state is developed using these parameters and a simple tensor transformation. The model of the faulty SCIM is derived by and additional tensor transformation of the healthy SCIM model in Section 5. An experimental validation of the proposed approach is carried on in Section 6 using a commercial SCIM with different cage faults. Finally, Section 7 presents the conclusions of this work.

## 2. Analytical Model of the SCIM

**Figure 1:**
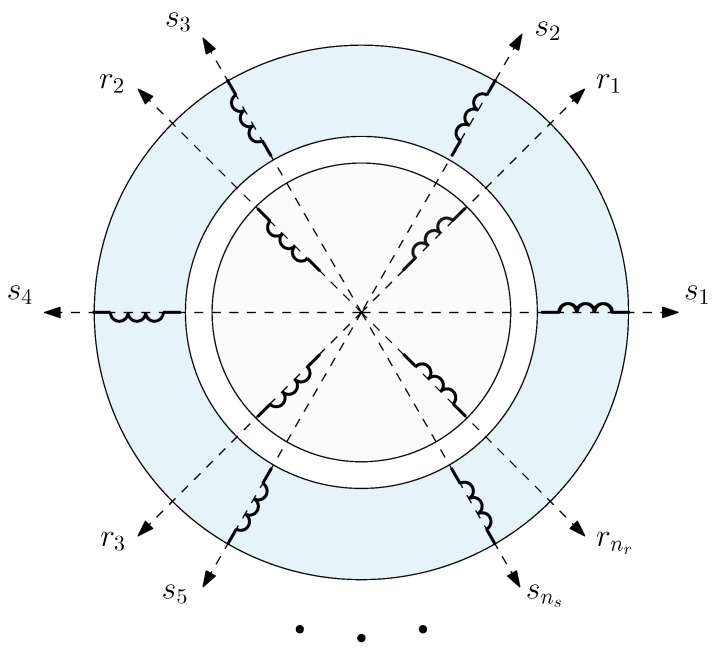
Coordinate system of the IM with an electrical axis rigidly connected to each phase, static in the case of the $n_{s}$ stator windings and moving with the rotor in case of the $n_{r}$ rotor windings.

Let us consider a generic IM with $n_{s}$ stator windings and $n_{r}$ rotor windings, with a total number of windings $n=n_{s}+n_{r}$. A holonomic reference frame [26] will be used in this paper, with an electrical axis rigidly connected to each winding, as seen in Figure 1. Therefore, the axes attached to the $n_{s}$ windings phases ($s_{1},s_{2},\ldots ,s_{n_{s}},$ in Figure 1) will be static, and those attached to the $n_{r}$ rotor windings ($r_{1},r_{2},\ldots ,r_{n_{r}}$, in Figure 1) will move with the rotor.

The *n* winding currents are the components of the current tensor i in this reference frame, that is

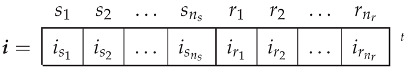
(1)
where the superscript ${}^{t}$ stands for the transpose operator. For easy of notation, if the axes information in (1) is omitted, then only the tensor components will be indicated as $i={\left[is_{1},is_{2},\ldots ,is_{n_{s}},ir_{1},ir_{2},\ldots ,ir_{n_{r}}\right]}^{t}$.

The tensor equations of voltage and torque of the IM in this coordinate system are [26,31]

(2) $$\begin{array}{cccc} • & \;\mathrm{Equation}\;\mathrm{of}\;\mathrm{voltage}: & \; & e=Ri+\frac{d\varphi }{dt}\\ • & \;\mathrm{Equation}\;\mathrm{of}\;\mathrm{torque}: & \; & T=J\frac{d\dot{\theta }}{dt}-\frac{1}{2}i^{t}\frac{dL}{d\theta }i \end{array}$$

Besides the current tensor, i, the quantities that appear in (2) are the following ones:

- e is the voltage tensor. Its *n* components are the instantaneous terminal voltages applied to each winding $e={\left[e_{s_{1}},e_{s_{2}},\ldots ,e_{s_{n_{s}}},e_{r_{1}},e_{r_{2}},\ldots ,e_{r_{n_{r}}}\right]}^{t}$.
- φ is the flux linkage tensor. Its *n* components are the instantaneous flux linkages of each winding $\varphi \;=\;{\left[{\varphi_{s}}_{1},{\varphi_{s}}_{2},\ldots ,{\varphi_{s}}_{n_{s}},{\varphi_{r}}_{1},{\varphi_{r}}_{2},\ldots ,{\varphi_{r}}_{n_{r}}\right]}^{t}$.
- R is the resistance tensor. It is a diagonal tensor, with $n^{2}$ components, whose elements are the resistances of the windings.
- L is the inductance tensor. It is a dyadic tensor, whose $n^{2}$ components are the self and mutual inductances of the windings. It relates the current and flux linkage tensors as φ=L·i.
- The rest of the terms that appear in (2) are the instantaneous applied shaft torque *T*, the rotor instantaneous angle θ and speed $\dot{\theta }$, and the moment of inertia of the rotor *J*.

The inductance tensor L can be expressed as the sum of two components, one with the inductances corresponding to the main flux linkages, the main inductance matrix $L_{\mu }$, and other with the leakage inductances $L_{\sigma }$, as

(3) $$L=L_{\mu }+L_{\sigma }$$

End turns, end rings, and slots leakage inductances, included in the diagonal $L_{\sigma }$ matrix, need to be pre-calculated, as usual in the technical literature, where explicit expressions for these inductances can be found in [32,33,34]. Only the analytical computation of $L_{\mu }$ in (3) will be carried out in this work.

**Figure 2:**
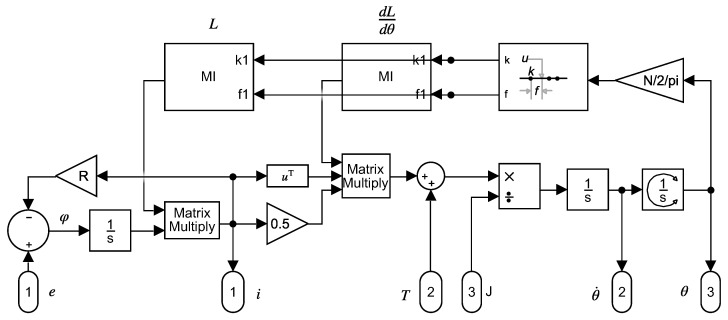
Analytical model that implements (2) in Simulink.

A Simulink model that implements (2) is shown in Figure 2. As seen in Figure 2, the mutual inductances between the stator and rotor windings depend on the rotor position, and must be updated at each step of the simulation.

The reference frame used for the analytical model of the IM in Figure 1 is not unique. If the current tensor is expressed in a different reference frame, its new components $i^{\prime }$ would be different than the old ones, i. Nevertheless, if the matrix C of the coordinate transformation is given, then the relation between the old components and the new ones is given by

(4) $$i=C\cdot i^{\prime }$$

and the transformation law of the rest of tensors e, R and L is given, applying tensor algebra, by

(5) $$\begin{array}{ccc} e^{\prime } & = & C^{t}\cdot e\\ \varphi^{\prime } & = & C^{t}\cdot \varphi \\ L^{\prime } & = & C^{t}\cdot L\cdot C\\ R^{\prime } & = & C^{t}\cdot R\cdot C \end{array}$$

In the case that the new reference frame is also holonomic, with all the electrical axes rigidly attached to the windings, then (2) remains valid, just substituting the old tensors by the new ones [26], as

(6) $$\begin{array}{cccc} • & \;\mathrm{Equation}\;\mathrm{of}\;\mathrm{voltage}: & \; & e^{\prime }=R^{\prime }i^{\prime }+\frac{d\varphi^{\prime }}{dt}\\ • & \;\mathrm{Equation}\;\mathrm{of}\;\mathrm{torque}: & \; & T=J\frac{d\dot{\theta }}{dt}-\frac{1}{2}i^{\prime }{}^{t}\frac{dL^{\prime }}{d\theta }i^{\prime } \end{array}$$

In this work, only holonomic reference frames, with all the electrical axis rigidly attached to the windings, will be used. Therefore, (6) will remain valid for all the reference frames used for modelling the SCIM both in healthy and faulty conditions.

The parameters of the model of Figure 2, both in healthy and faulty conditions, are obtained in this work using simple tensor transformations based on constructive data and on the resistances and leakage inductances of stator windings, rotor bars and end-ring segments. Most of these basic parameters can be found in the technical data provided by the manufacturer of the IM, as in the case of the machine used for the experimental tests in this work. If these specifications are not available, they can be estimated using offline [35,36,37] or online parameter estimation techniques [38]. A comprehensive review of these techniques can be found in [39]. Recently, artificial intelligence (AI) methods for parameter estimation have been proposed in [40], or using differential evolution algorithms [41]. Additionally, IM parameters change with temperature, frequency, and saturation, which has not been considered in the model used in with work.

It is worth mentioning that the model of Figure 2 is a dynamical one. Therefore, it can be applied to IMs working in stationary regime, or under transient conditions, as in [42]. Besides, being an analytical model, it is very fast, and can be run in real time. This opens the possibility of using it not only for fault diagnosis of IMs, which is the focus of this work, but also for speed estimation in sensorless control systems [43], or for reducing torque oscillations produced by space harmonics [44,45], among many other technical applications.

## 3. Primitive Reference Frame of the SCIM

Let us consider an SCIM with $n_{s}$ stator windings and a cage with $n_{b}$ rotor bars. Instead of deriving directly the inductance and resistance matrices of the rotor loops and stator windings, a simpler reference frame will be used as starting point, the primitive reference frame, as proposed in [26]. The matrices obtained in this simpler reference frame will be converted to the final ones using easy tensor transformations.

**Figure 3:**
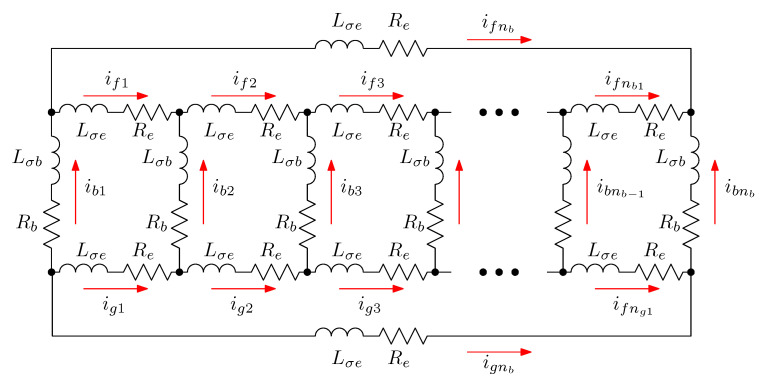
Primitive reference frame used for the rotor cage, with $n_{b}$ bars. Each bar and each end ring segment have a rigidly attached coordinate axis. The bar and end ring segment currents are the components of the current tensor in this frame. The bars are coupled to each other and to the stator currents through their mutual inductances (not shown in this circuit). On the contrary, the end ring segments do not couple with the other windings through mutual inductances.

Following the method of tensor analysis proposed by Kron in [26], the rotor cage network is modelled first by considering bar and end ring currents as independent variables, shown in Figure 3. The actual rotor cage parameters will be obtained from this primitive network by using a transformation matrix that represents the connections between those elements, and applying (5).

The characteristics of the primitive reference frame represented in Figure 3 are the following ones:

- The stator electrical axes are attached to the $n_{s}$ stator windings (usually three for industrial SCIMs). The unit vectors along these axes will be denoted as $s_{1},s_{2},\ldots ,s_{n_{s}}$, and the components of the current tensor will be denoted as $i_{s_{1}},i_{s_{2}},\ldots ,i_{s_{n_{s}}}$. All the stator windings are considered to have the same resistance $R_{s}$ and leakage inductance $L_{\sigma s}$.
- Each bar of the rotor cage has attached rigidly an electrical axis. The unit vectors along these axes will be denoted as $b_{1},b_{2},\ldots ,b_{n_{b}}$, and the components of the current tensor will be denoted as $i_{b_{1}},i_{b_{2}},\ldots ,i_{b_{n_{b}}}$. All the bars are considered to have equal resistance $R_{b}$ and leakage inductance $L_{\sigma b}$.
- Each end ring segment of the cage has attached rigidly an electrical axis. The unit vectors along the axes of the segments of one end ring will be denoted as $f_{1},f_{2},\ldots ,f_{n_{b}}$, and $g_{1},g_{2},\ldots ,g_{n_{b}}$ for the opposite end ring. The components of the current tensor along these axes will be denoted as $i_{f_{1}},i_{f_{2}},\ldots ,i_{f_{nb}}$ for the segments of one end ring and $i_{g_{1}},i_{g_{2}},\ldots ,i_{g_{n_{b}}}$ for the opposite one. All the end ring segments are considered to have equal resistance $R_{e}$ and leakage inductance $L_{\sigma e}$.

**Figure 4:**
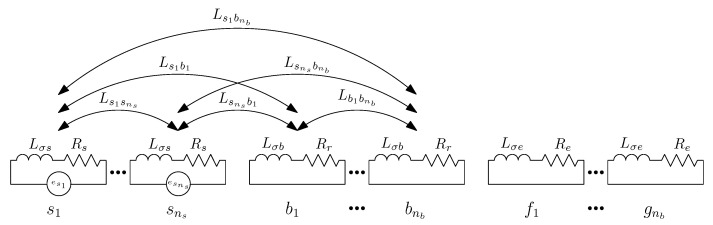
Primitive reference frame of the SCIM, found by removing all interconnections between the windings, and short circuiting each. The arrows show the mutual impedances between stator windings and cage bars. The end ring segments do not couple with the other windings through mutual impedances.

Therefore, the primitive network of the SCIM is built by removing all interconnections between the windings and short circuiting each, as shown in Figure 4.

In the primitive reference frame of Figure 4, the current tensor $i_{p}$ has ($n_{s}+3\cdot n_{b}$) components:

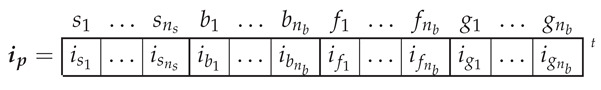
(7)

In this primitive reference frame, the voltages of the stator windings are considered to be independent variables, while the voltages applied to the rotor windings are zero. That is, the voltage tensor is given by

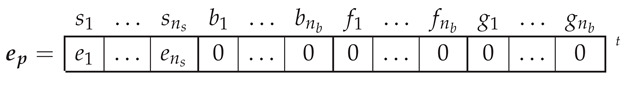
(8)

### 3.1. Resistance Tensor of the SCIM in the Primitive Reference Frame

The resistance tensor in the primitive reference frame $R_{p}$ is a diagonal tensor, of size $\left(n_{s}+3\cdot n_{b}\right)\times \left(n_{s}+3\cdot n_{b}\right)$, with the following components (from now on, the matrix elements with a zero value will be left blank, for easy of presentation):

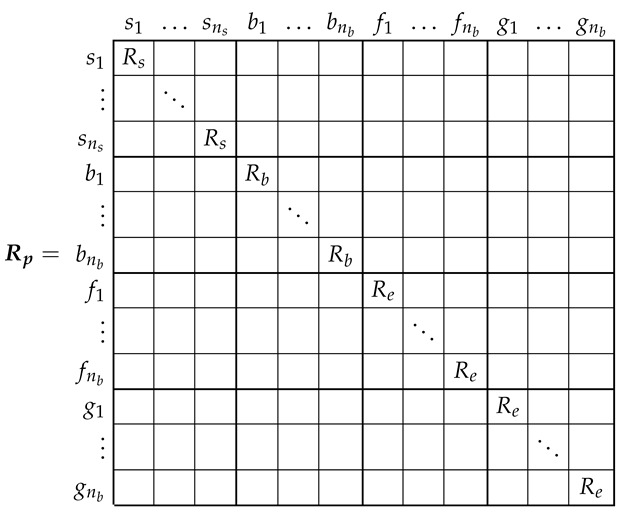
(9)

### 3.2. Leakage Inductance Tensor of the SCIM in the Primitive Reference Frame

The leakage inductance tensor in this reference frame, $L_{p\sigma }$ (3), is also a diagonal tensor, of size $\left(n_{s}+3\cdot n_{b}\right)\times \left(n_{s}+3\cdot n_{b}\right)$, with the following components:

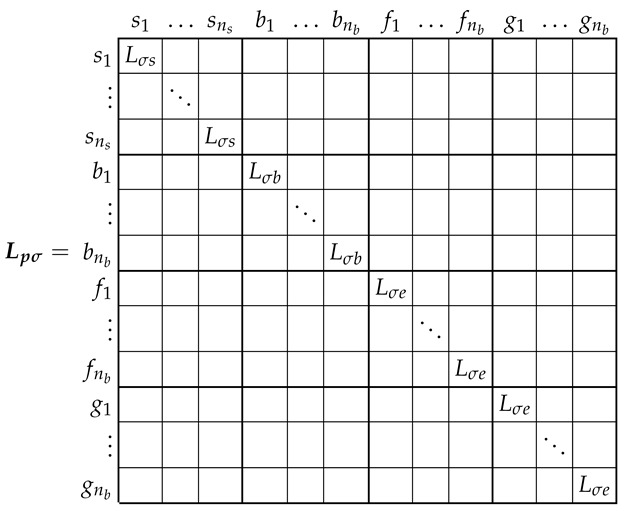
(10)

It is worth mentioning that, in the primitive reference frame, the resistance (9) and the leakage inductance (10) matrices are diagonal ones.

### 3.3. Main Inductance Tensor of the SCIM in the Primitive Reference Frame

The main inductance tensor in this reference frame, corresponding to the main flux linkages, $L_{p\mu }$ (3), is a dyadic tensor of size $\left(n_{s}+3\cdot n_{b}\right)\times \left(n_{s}+3\cdot n_{b}\right)$, with the following components:

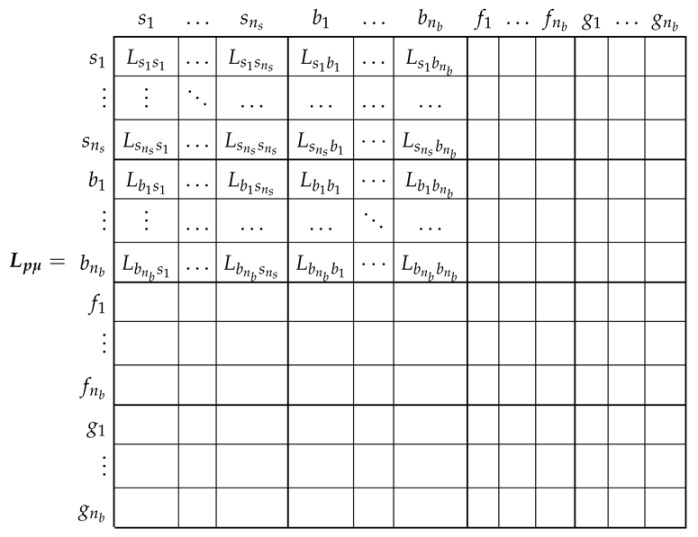
(11)

As displayed in $L_{p\mu }$ (11), the mutual inductances between the end ring segments and the rest of the windings due to the main flux linkages are zero, because their only flux linkages are the leakage ones. As for the rest of the components of (11), they depend on the actual stator and rotor winding configurations, and, besides, the mutual inductances between stator and rotor windings also depend on the rotor angular position. Among the many available methods in the technical literature for obtaining their values (FEM, WFA, etc.), in this work the winding tensor approach has been selected, which is described briefly in the next subsection.

### 3.4. Computation of the Main Inductances of the SCIM Using the Winding Tensor Approach

Neglecting the iron saturation and losses, mutual inductances depend only on the geometry of the system [46]. Only the radial component of the main flux that crosses the smooth air gap is considered in this work, and the iron permeability has been considered as infinite. The analytical computation of mutual inductances considering also the tangential component of the flux can be found in [25], and with non-uniform air-gap in [29]. A higher precision can be achieved using numerical methods, such as those based in FEM [20,47], but at the cost of an increased computing complexity. Nevertheless, the simple, analytical approach followed in this work has proven to be able to reproduce correctly the fault harmonics of the cage fault, while keeping a low computational burden.

A simplified computation of the self and mutual inductances between the IM windings can be made assuming a sinusoidal distribution of their MMFs, thereby neglecting the spatial harmonics generated by the windings distribution. This simplification hampers the use of the analytical model presented in Figure 2 for fault diagnosis, because in case of a fault there are complex interactions between spatial and time harmonics that can not be reproduced by such a simplified model. In [26], the calculation of self and mutual winding inductances with spatial harmonics was made by first establishing the mutual inductance between two elementary coils, in different relative positions, and then transforming it into winding inductances using a transformation matrix, as in (5). In [29], a similar procedure was presented, but using a single conductor instead of a coil as the most basic unit, which avoids the need to establish a different winding function for each relative position between two coils.

**Figure 5:**
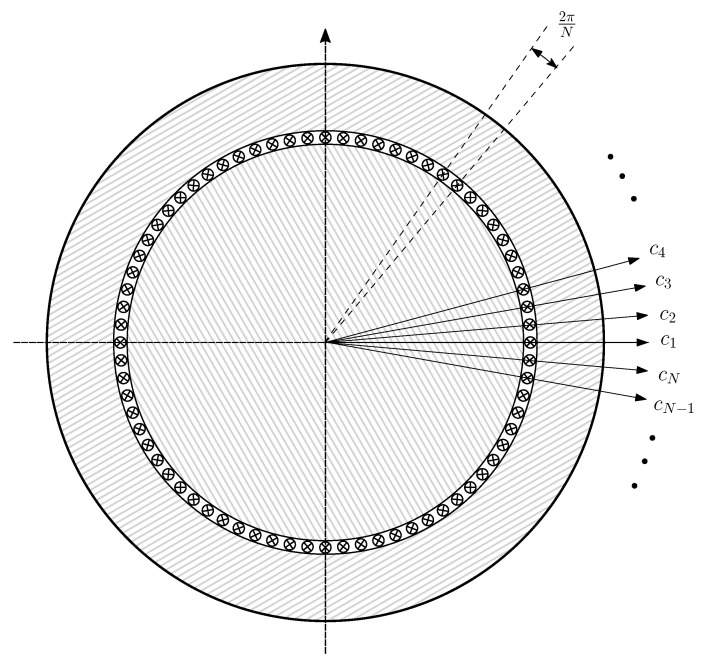
Reference frame constituted by *N* independent conductors placed in the air gap. The *N* components of the air gap current tensor in this system, $i_{c}$, are the currents through each elementary conductor.

For using the conductor as the most basic unit, a reference frame is established by considering that the circular air gap is evenly divided into *N* segments, and that each of them is filled with an elementary conductor, located in the air gap zone, with an electrical axis attached to it (see Figure 5). In [25], two layers of conductors have been considered instead, one placed on the inner stator surface and the other one placed on the outer rotor surface. The maximum spatial harmonic of the winding that can be represented in this reference frame is N/2, and, therefore, a high value of *N* is selected (N=3600 in [29]).

In this reference frame, the air gap current tensor components, $i_{c}$, are the currents through each elementary conductor.

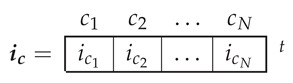
(12)

In the reference frame of Figure 5, the main inductance tensor, $L_{c\mu }$, is a N×N dyadic tensor, given by

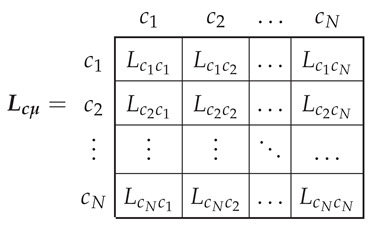
(13)
whose component (i,j), $L_{c_{i}c_{j}}$, is the mutual partial inductance [25] between the conductors placed at positions $\left(i-1\right)\cdot \frac{2\pi }{N}$ and $\left(j-1\right)\cdot \frac{2\pi }{N}$, with i,j=1,2,…,N. In case of an IM with uniform air gap length, as represented in Figure 5, and considering that the air gap is small compared to its radius, $L_{c_{i}c_{j}}$ depends only on the angular separation between conductors *i* and *j*, and is given by [42]

(14) $$L_{c\mu }\left(i,j\right)=L_{c_{i}c_{j}}=\frac{\mu_{0}\cdot l\cdot r\cdot \pi }{g}\cdot {\left(\frac{1}{2}-\frac{\left|i-j\right|}{N}\right)}^{2}$$

where $\mu_{0}=4\pi 10^{-7}$, *l* is the effective length of the stator bore, *r* is the radius at the center of the air gap, and *g* is the air gap length.

The expression of the mutual inductance between conductors $L_{c\mu }$ has also been obtained considering an analytic two dimensional field analysis in [25,48], or a numerical model in [47]. Besides, it has been obtained in the case of a non-uniform air gap due to rotor eccentricity in [29,49].

From (14), the components of $L_{c\mu }$ are the same for every IM, except for the scaling factor $\frac{\mu_{0}\cdot l\cdot r\cdot \pi }{g}$, which depends only on the geometrical dimensions of the machine *l*, *r* and *g*. Besides, $L_{c\mu }$ is a circulant, symmetrical, matrix, where every column is obtained by shifting one position the preceding column.

The relation between the old currents $i_{c}$ (12), and the new ones $i_{p}$ (7) can be formulated using a $\left(N\times \left(n_{s}+3n_{b}\right)\right)$ winding tensor $C_{c}$ as (4)

(15) $$i_{c}=C_{c}\cdot i_{p}$$

where

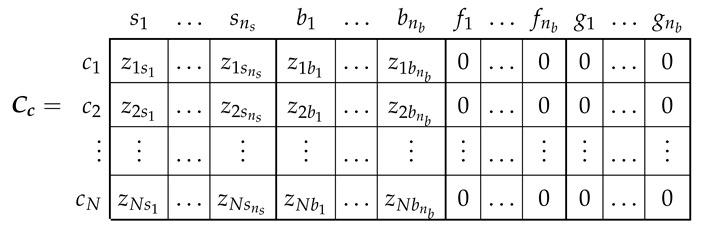
(16)

The winding tensor $C_{c}$ (16) represents the connections between the conductors of each winding. Its (i,j) element contains the number of conductors $z_{ij}$ of winding *j* contained in the angular interval 2π/N (Figure 5), centred at $\left(i-1\right)\cdot \frac{2\pi }{N}$, with the corresponding sign according to the direction of the current. For example, the distribution of the conductors along a stator slot that contains $Z_{slot}$ conductors of a given stator winding, and has a slot opening equal to *b*, would be a constant value of $z_{sc}=Z_{slot}/b\cdot \left(2\pi r_{s}\right)/N$ air gap conductors per interval along the slot opening (with its corresponding sign), where $r_{s}$ is the inner radius of the stator bore. In this way, the effects of the slot width or the rotor bar inclination can be represented with up to N/2 spatial harmonics. In the case of the rotor end rings, as they have no conductors in the air gap, the corresponding columns in $C_{c}$ ($e_{1},\ldots ,e_{n_{b}},f_{1},\ldots ,f_{n_{b}}$) are zero. These columns have been maintained in (16) for the sake of completeness.

The main inductance tensor of the windings in the reference frame of Figure 3, $L_{p\mu }$ (11), can be obtained from the main inductance tensor of the conductors in the reference frame of Figure 5, $L_{c\mu }$ (13), applying the routine tensor algebra transformation (4) with the winding tensor $C_{c}$ (16), as

(17) $$L_{p\mu }={C_{c}}^{t}\cdot L_{c\mu }\cdot C_{c}$$

The winding tensor $C_{c}$ (16) must be obtained for the *N* possible angular positions of the rotor ($\theta_{k}=\left(k-1\right)\cdot \frac{2\pi }{N}$, with k=1,…,N). Nevertheless, the columns of $C_{c}$ corresponding to the $n_{s}$ stator windings do not depend on the rotor position, and the columns of $C_{c}$ corresponding to the rotor windings for a given rotor position $\theta_{k}$ are the same as the columns defined with the rotor at the origin ($\theta_{0}=0$), but circularly shifted *k* positions.

In (16), no restrictions are imposed on the connections of the conductors of each winding, which can be arbitrarily complex, as in the case of asymmetrical windings (turn-to-turn short circuits, etc.). Nevertheless, in case of a healthy machine, the configuration of all the stator and rotor windings are the same, respectively. Therefore, the column of $C_{c}$ corresponding to the *k*th stator winding ($s_{k}$) is equal to the column of the first stator winding ($s_{1}$), but circularly shifted $k\cdot N/n_{s}$ positions. The same applies to the rotor windings, but in this case the circular shift is $k\cdot N/n_{b}$ positions. In this particular case, the computation of (16) can be performed in a very fast way using the convolution theorem, based on the the circulant properties of matrix $L_{c\mu }$, as presented in [42].

## 4. Analytical Model of the SCIM in Healthy State

In Section 3, a primitive reference frame has been used, considering the bar and the end ring segment currents as independent variables. The number of rotor equations of the SCIM in this reference frame is very high, equal to the sum of the number of rotor bars and twice the number of end-ring segments. To reduce it, rotor loop equations are established in this section, taking into account the connections between the cage components (bars and end ring segments), using a connection tensor. The parameters of the SCIM using rotor loop currents will be found by applying routine tensor algebra (5).

### 4.1. Connection Tensor for the Rotor Bars and End Ring Segments

**Figure 6:**
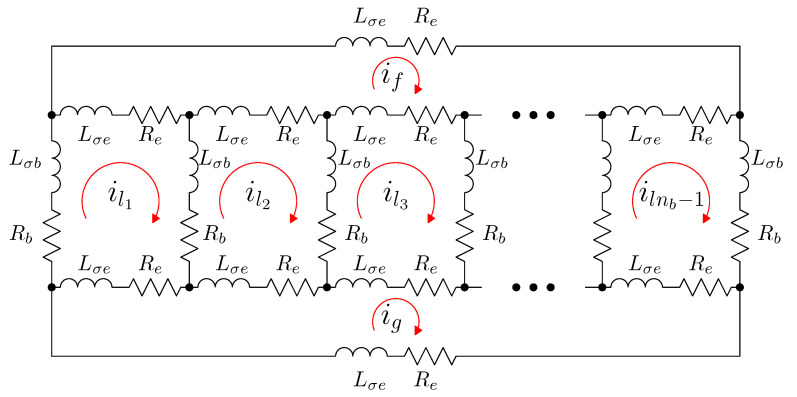
Rotor loops in a squirrel cage rotor of $n_{b}$ bars. There are $n_{b}-1$ rotor loops, formed by two consecutive bars, coupled to each other and to the stator windings through their mutual inductances (not displayed in this schema). Besides, there are two end ring loops, which do not couple with any other windings through mutual inductances.

The new reference frame consists of attaching an electrical axis to each rotor loop, which is constituted by two adjacent rotor bars and their connecting end ring segments, as displayed in Figure 6.

Therefore, in the reference frame of Figure 6, the current tensor i will have ($n_{s}+n_{b}+1$) components, the currents in the $n_{s}$ stator windings, the $n_{b}-1$ rotor loops and the two end ring loops:

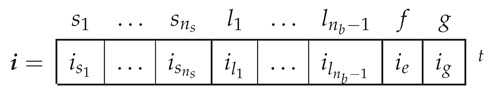
(18)

The relation between the primitive network currents $i_{p}$ (7), and the new ones i (18) can be formulated using a $\left(\left(n_{s}+3n_{b}\right)\times \left(n_{s}+n_{b}+1\right)\right)$ connection tensor $C_{p}$, with the aid of Kirchoff’s laws, as

(19) $$i_{p}=C_{p}\cdot i$$

The connection tensor $C_{p}$ can be established by direct comparison of Figure 3 and Figure 6, just indicating the connections between the individual bars and end ring segments forming each rotor loop as: 
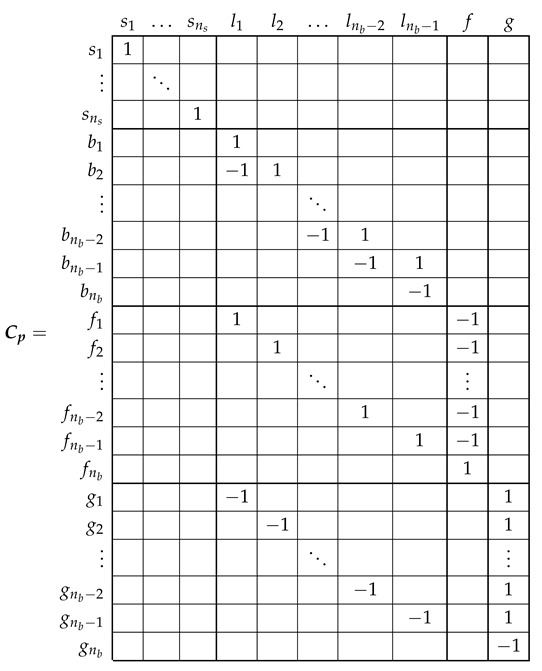
(20)

Using the connection tensor $C_{p}$ in (4), the tensors’ components in this new reference frame are obtained directly from their components in the primitive reference frame as

(21) $$\begin{array}{ccccccccc} \mathrm{voltage}\;\mathrm{tensor} & & e & = & {C_{p}}^{t} & \cdot & e_{p} & & \\ \mathrm{resistance}\;\mathrm{tensor} & & R & = & {C_{p}}^{t} & \cdot & R_{p} & \cdot & C_{p}\\ \mathrm{leakage}\;\mathrm{inductance}\;\mathrm{tensor} & & L_{\sigma } & = & {C_{p}}^{t} & \cdot & L_{p\sigma } & \cdot & C_{p}\\ \mathrm{main}\;\mathrm{inductance}\;\mathrm{tensor} & & L_{\mu } & = & {C_{p}}^{t} & \cdot & L_{p\mu } & \cdot & C_{p} \end{array}$$

For example, the resistance tensor R (rotor loop resistances in Figure 6) is obtained, applying (21), as

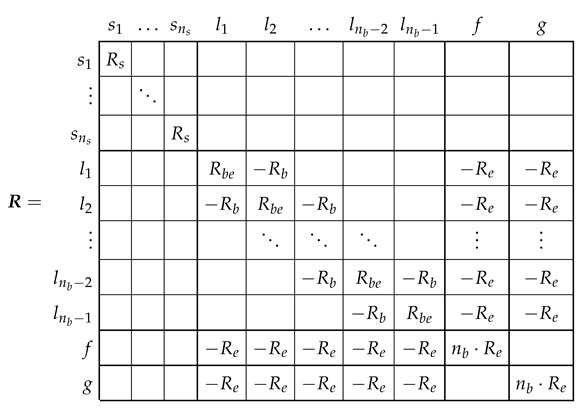
(22)
which checks with the expression given in [29], with $R_{be}=2\left(R_{b}+R_{e}\right)$. That is, the careful derivation of the circuit equations of the network of Figure 6 has been replaced by routine laws of tensor transformations applied to the much simpler primitive network parameters, just the diagonal resistance matrix (9), using a transformation matrix whose elements are ones, minus ones and zeros (20).

### 4.2. Connection Tensor for the Stator Windings

In the primitive reference frame of Section 3, the stator currents have been considered as independent variables. In this section, the connections between the stator windings are taken into account, using a connection tensor, and the actual parameters of the SCIM will be found by applying routine tensor algebra (5). The stator currents can be considered as independent variables if each winding is fed with an independent power source, or if they are connected in star configuration, fed from a power line with a distributed neutral connected to the neutral point of the star. In any other case, the connection tensor of the stator windings must be applied to the primitive tensors to obtain the actual SCIM ones.

For example, in case of a star connection of the stator windings, fed from a power line without distributed neutral, the following constraint of the stator currents applies:

(23) $$\sum_{i=1}^{n_{s}}i_{s_{i}}=0\Rightarrow i_{s_{n_{s}}}=-\sum_{i=1}^{n_{s}-1}i_{s_{i}}$$

This constraint reduces the number of independent stator currents by one. Therefore, the current tensor (18) will have $\left(n_{s}+n_{b}\right)$ components, the currents in the $n_{s}-1$ stator windings, the $n_{b}-1$ rotor loops and the two end ring loops:

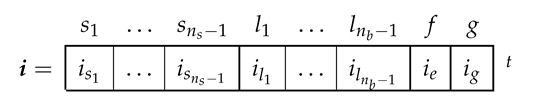
(24)
and the connection tensor $C_{p}$ (20) becomes: 
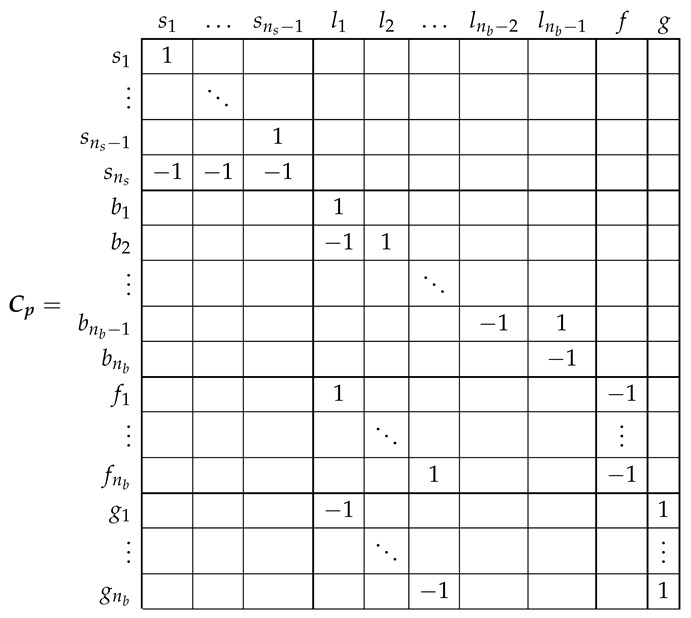
(25)

For ease of presentation, in this work it will be assumed that the stator currents are independent variables, and, therefore, the connection tensor (20) will be used for obtaining the SCIM parameters, and the voltage tensor (8) will not be modified by the connection tensor $C_{p}$. That is, $e=e_{p}$ in (21).

### 4.3. Voltage and Torque Equations of the SCIM with a Healthy Rotor Cage

As seen in (21), the parameters of the SCIM with a healthy cage in the rotor loop frame, Figure 6, can be obtained directly from the parameters of the SCIM in the primitive reference frame, Figure 6, where they adopt their simplest form (diagonal matrices for the resistance and leakage inductance tensors, partial inductances between single rotor bars and stator windings). The new parameters have been found by routine tensor transformations, using a simple connection matrix $C_{p}$, whose element are ones, minus ones and zeros, which just reflect the connections between bars and end ring segments in the healthy rotor cage (20). As this transformation is holonomic (the new rotor axes remain rigidly attached to the rotor windings), the voltage and torque equations (2), being a tensorial equation, remain valid, just replacing the old by the new, transformed SCIM tensors.

## 5. Analytical Model of the SCIM with Rotor Cage Faults

In this section, the parameters of a SCIM with rotor cage faults is obtained from the resistance and inductance tensors of the healthy machine, by defining a transformation tensor that takes into account each type of fault, and applying routine tensor transformation laws. Three cases will be considered next: a cage with a broken bar, a cage with two non-consecutive rotor bars, and a cage with a broken end ring segment. Other faults such as non-consecutive broken bars, or the combined breakage of end ring segments and rotor bars can be treated in a similar way.

### 5.1. Analytical Model of the SCIM with a Broken Rotor Bar

**Figure 7:**
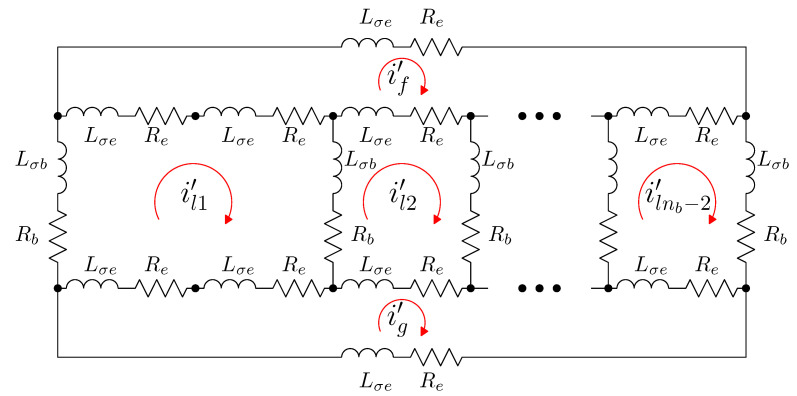
Rotor loops in a squirrel cage rotor of $n_{b}$ bars with a single broken bar ($b_{2}$). It is similar to the circuits in a healthy rotor cage shown in Figure 6, but now the first rotor loop is formed by two non-consecutive bars ($b_{1}$ and $b_{3}$).

The rotor network of an SCIM with a single broken rotor bar ($b_{2}$ in this example) can be established as depicted in Figure 7. This electrical network is derived from the healthy rotor cage shown in Figure 6, but now the first rotor loop (which contains the broken bar $b_{2}$) is formed by two non-consecutive rotor bars ($b_{1}$ and $b_{3}$).

In the reference frame of Figure 7, the current tensor $i^{\prime }$ will have ($n_{s}+n_{b}$) components, the currents in the $n_{s}$ stator windings, the $n_{b}-2$ rotor loops and the two end ring loops.

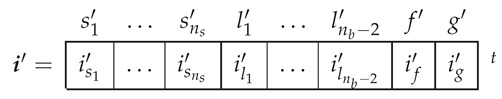
(26)

The transformation tensor that relates the currents in the healthy and in the faulty reference frame, $C_{b_{2}}$, so that $i=C_{b_{2}}\cdot i^{\prime }$, can be established by direct comparison of Figure 3 and Figure 7, as: 
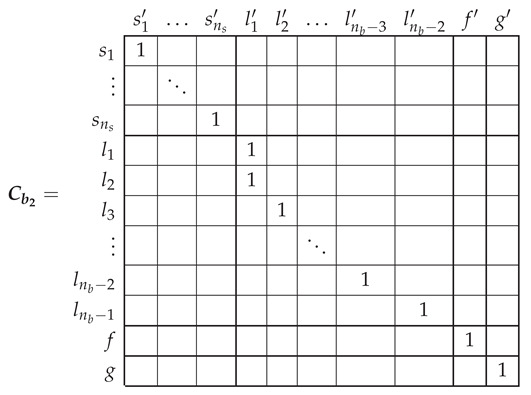
(27)

That is, what $C_{b_{2}}$ reflects is simply that the effect of a broken bar in Figure 7 can be represented by equating the currents in the two rotor loops that contain the missing bar, in this case loops $l_{1}$ and $l_{2}$. As the transformation tensors of the current form a group, their combined effect is obtained by a simple product. Therefore, the final tensors of the SCIM with a broken bar of Figure 7 are obtained, using both connecting tensors $C_{p}$ and $C_{b_{2}}$ as

(28) $$\begin{array}{ccccccccc} \mathrm{resistance}\;\mathrm{tensor} & & R^{\prime } & = & {\left(C_{p}\cdot C_{b_{2}}\right)}^{t} & \cdot & R_{p} & \cdot & \left(C_{p}\cdot C_{b_{2}}\right)\\ \mathrm{leakage}\;\mathrm{inductance}\;\mathrm{tensor} & & L_{\sigma }^{\prime } & = & {\left(C_{p}\cdot C_{b_{2}}\right)}^{t} & \cdot & L_{p\sigma } & \cdot & \left(C_{p}\cdot C_{b_{2}}\right)\\ \mathrm{main}\;\mathrm{inductance}\;\mathrm{tensor} & & L_{\mu }^{\prime } & = & {\left(C_{p}\cdot C_{b_{2}}\right)}^{t} & \cdot & L_{p\mu } & \cdot & \left(C_{p}\cdot C_{b_{2}}\right) \end{array}$$

### 5.2. Analytical Model of the SCIM with Two Non-Consecutive Broken Rotor Bars

**Figure 8:**
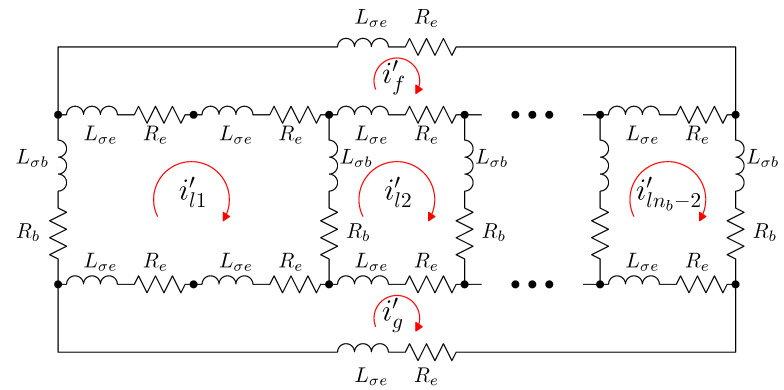
Rotor loops in a squirrel cage rotor of $n_{b}$ bars with two non-consecutive broken bars ($b_{2}$ and $b_{4}$). It is similar to the circuits in a healthy rotor cage shown in Figure 6, but now the the first two rotor loops contain non-consecutive broken bars.

The electrical rotor network of a SCIM with two non-consecutive broken rotor bars ($b_{2}$ and $b_{4}$ in this example) can be established as depicted in Figure 8. This network is similar to the healthy rotor cage shown in Figure 6, but now the first two rotor loops contain the non consecutive broken bars.

In the reference frame of Figure 8, the current tensor $i^{\prime }$ will have ($n_{s}+n_{b}-1$) components, the $n_{s}$ stator currents, the $n_{b}-3$ rotor loop currents and the two end ring currents.

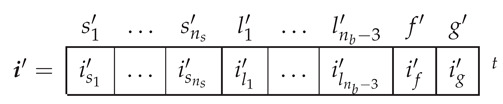
(29)

The transformation tensor that relates the currents in the healthy (18) and in the faulty (29) reference frames, $C_{b_{2}b_{4}}$, so that $i=C_{b_{2}b_{4}}\cdot i^{\prime }$, can be established by direct comparison of Figure 3 and Figure 7, as:

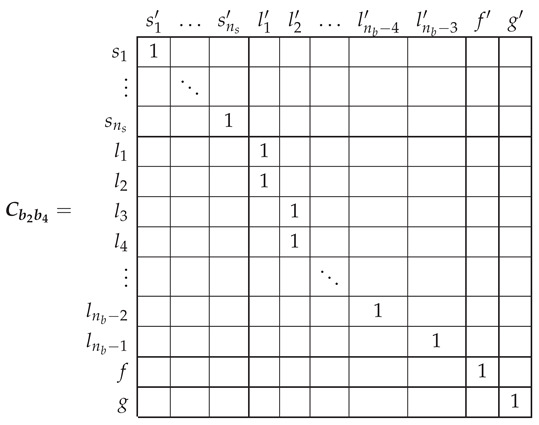
(30)

That is, what $C_{b_{2}b_{4}}$ reflects is simply that the effect of two broken bars in Figure 8 can be represented by making equal the currents in the two rotor loops that contain each missing bar. As the transformation tensors of the current form a group, their combined effect is obtained by a simple product. Therefore, the final tensors of the SCIM with two non-consecutive broken bars of Figure 8 are obtained, using both connecting tensors $C_{p}$ and $C_{b_{2}b_{4}}$ as

(31) $$\begin{array}{ccccccccc} \mathrm{resistance}\;\mathrm{tensor} & & R^{\prime } & = & {\left(C_{p}\cdot C_{b_{2}b_{4}}\right)}^{t} & \cdot & R_{p} & \cdot & \left(C_{p}\cdot C_{b_{2}b_{4}}\right)\\ \mathrm{leakage}\;\mathrm{inductance}\;\mathrm{tensor} & & L_{\sigma }^{\prime } & = & {\left(C_{p}\cdot C_{b_{2}b_{4}}\right)}^{t} & \cdot & L_{p\sigma } & \cdot & \left(C_{p}\cdot C_{b_{2}b_{4}}\right)\\ \mathrm{main}\;\mathrm{inductance}\;\mathrm{tensor} & & L_{\mu }^{\prime } & = & {\left(C_{p}\cdot C_{b_{2}b_{4}}\right)}^{t} & \cdot & L_{p\mu } & \cdot & \left(C_{p}\cdot C_{b_{2}b_{4}}\right) \end{array}$$

### 5.3. Analytical Model of the SCIM with a Broken End Ring Segment

**Figure 9:**
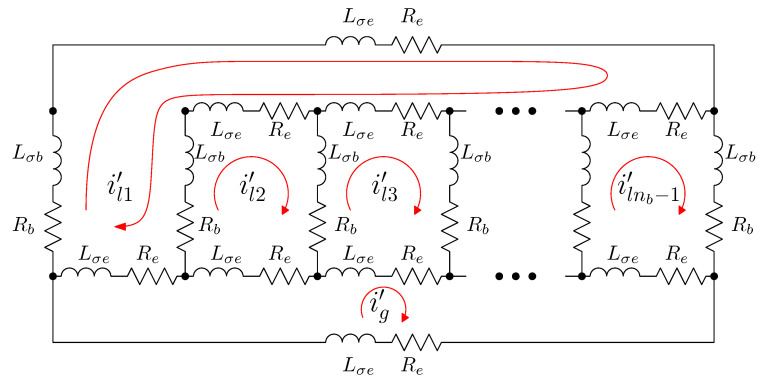
Rotor loops in a squirrel cage rotor of $n_{b}$ bars with a single broken end ring segment ()$f_{1}$). It is similar to the circuits in a healthy rotor cage shown in Figure 6, but now the first rotor loop includes the whole end ring loop.

The rotor network of a SCIM with a broken end ring segment ($f_{1}$ in this example) can be established as depicted in Figure 9. This circuit is similar to the circuit of a healthy rotor cage shown in Figure 6, but now the first rotor loop (which contains the broken end segment ) includes the whole end ring loop.

In the reference frame of Figure 9, the current tensor $i^{\prime }$ will have ($n_{s}+n_{b}$) components, the $n_{s}$ stator currents, the $n_{b}-1$ rotor loop currents and the healthy end ring current.

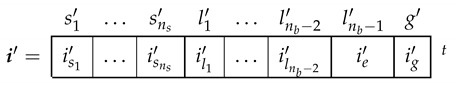
(32)

The transformation tensor that relates the currents in the healthy (18) and in the faulty (32) reference frames, $C_{f_{1}}$, so that $i=C_{f_{1}}\cdot i^{\prime }$, can be established by direct comparison of Figure 3 and Figure 9, as: 
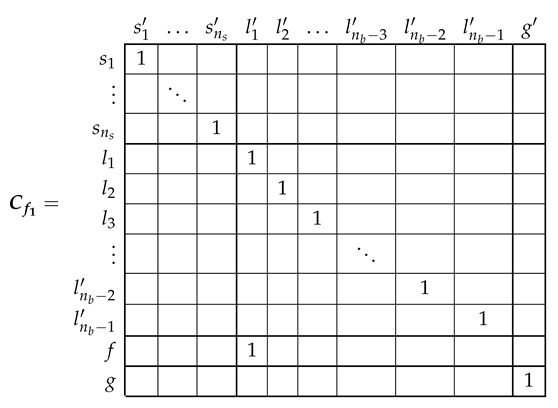
(33)

That is, what $C_{f_{1}}$ reflects is simply that the effect of a broken end ring segment in Figure 9 can be represented by making equal the current in the rotor loop that contain the missing end ring segment $f_{1}$, in this case loop $l_{1}$, and the current in the end ring loop. As the transformation tensors of the current form a group, their combined effect is obtained by a simple product. Therefore, the final tensors of the SCIM with a broken end ring segment of Figure 9 are obtained, using both connecting tensors $C_{p}$ and $C_{f_{1}}$ as

(34) $$\begin{array}{ccccccccc} \mathrm{resistance}\;\mathrm{tensor} & & R^{\prime } & = & {\left(C_{p}\cdot C_{f_{1}}\right)}^{t} & \cdot & R_{p} & \cdot & \left(C_{p}\cdot C_{f_{1}}\right)\\ \mathrm{leakage}\;\mathrm{inductance}\;\mathrm{tensor} & & L_{\sigma }^{\prime } & = & {\left(C_{p}\cdot C_{f_{1}}\right)}^{t} & \cdot & L_{p\sigma } & \cdot & \left(C_{p}\cdot C_{f_{1}}\right)\\ \mathrm{main}\;\mathrm{inductance}\;\mathrm{tensor} & & L_{\mu }^{\prime } & = & {\left(C_{p}\cdot C_{f_{1}}\right)}^{t} & \cdot & L_{p\mu } & \cdot & \left(C_{p}\cdot C_{f_{1}}\right) \end{array}$$

### 5.4. Voltage and Torque Equations of the SCIM with Cage Faults

As commented in Section 4.3, the parameters of the SCIM with a faulty cage can be obtained directly from the parameters of the SCIM in the primitive reference frame (the simplest one) by routine tensor transformations. Simple connection matrices $C_{p}$ (20), $C_{b_{2}}$ (27), $C_{b_{2}b_{3}}$ (30), and $C_{f_{1}}$ (33), whose elements are ones and zeros, which reflect the connections between bars and end ring segments in the faulty rotor cage, are used in this transformations. As the new rotor axes remain rigidly attached to the rotor windings, the voltage and torque Equation (2) remain valid, just using the transformed SCIM tensors. That is

(35) $$\begin{array}{c} \left\{\begin{array}{ccc} e^{\prime } & = & R^{\prime }i^{\prime }+L^{\prime }\frac{di^{\prime }}{dt}+i^{\prime }\frac{dL^{\prime }}{d\theta }\dot{\theta }\\ T & = & J\frac{d\dot{\theta }}{dt}-\frac{1}{2}i^{\prime }{}^{t}\frac{dL^{\prime }}{d\theta }i^{\prime } \end{array}\right. \end{array}$$

where $e^{\prime }=e$, because the stator windings voltages have not been changed (only rotor cage faults have been considered).

### 5.5. Analytical Model of the SCIM with Rotor Faults in Progress

In some cases, the faulty rotor bar or end ring segment are not completely broken, but their resistance or leakage reactance are different from normal values due to a fault in progress, such as an oxidation process [50]. In this case, the parameters of the faulty rotor part are simply adjusted in the corresponding diagonal element of the primitive resistance (9) or leakage inductance tensor (10). In this way, the proposed model can be applied to the prognosis of incipient broken rotor bars in an induction motor, as in [51], or half broken bars, as in [52]. Nevertheless, as [51] states, the deterioration of the bar is a highly non-linear process, and more advanced physical models, such as multi-physics finite-element analysis and fatigue testing would be necessary to establish a non-linear failure model for the prognosis of incipient broken bar faults.

## 6. Experimental Validation

The validation of the proposed approach has been carried out by the simulation and experimental tests of a commercial squirrel-cage induction motor, whose characteristics are given in Appendix A. The types of faults that have been used for the experimental validation of the proposed model are broken bars faults: a single broken bar, two consecutive broken bars, and two non-consecutive broken bars. In fact, major motor manufacturers have reported cases where the damaged bars appear randomly distributed around the rotor perimeter, indicating that the failure of non-adjacent bars is fairly common in large cage induction motors.

### 6.1. Experimental Setup

**Figure 10:**
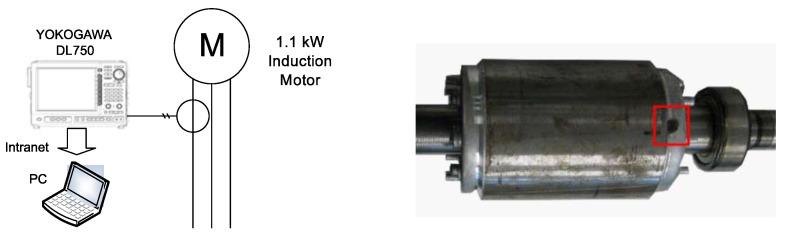
Experimental setup: measurement equipment (**left**), and rotor of the motor whose characteristics are given in Appendix A, with a broken bar (**right**).

The test equipment, displayed in Figure 10, consists of a current clamp, whose characteristics are given in Appendix B, a 200 pulse/revolution incremental encoder, a Yokogawa DL750 oscilloscope and a personal computer (Appendix C) connected to it via an intranet network. The broken bar fault has been artificially produced by drilling a hole in the selected bars, as shown in Figure 10.

The rotor cage faults are detected using the motor current signature analysis (MCSA) method. It is based on the identification in the current spectrum of the characteristic fault harmonic components generated by the fault, at frequencies given by

(36) $$f_{bb}=\left(1+2ks\right)f_{1}\;k=\pm 1,\pm 2,\pm 3\ldots$$

where *s* is the motor slip, $f_{1}$ is the supply frequency, and *k* is the harmonic number. For the tested and simulated motors, the rotor speed is the rated one, 1410 r.p.m, and the supply frequency is $f_{1}=50$ Hz. The main fault harmonics used for the diagnosis are those corresponding to a value k=±1 in (36): the lower side-band harmonic (LSH), with a frequency $f_{LSH}=\left(1-2s\right)f_{1}$, and the upper side-band harmonic (USH), with a frequency $f_{USH}=\left(1+2s\right)f_{1}$. In case of the tested and simulated motors s=0.06; therefore, $f_{LSH}=\left(1-2\times 0.06\right)\times 50=44$ Hz, and $f_{USH}=\left(1+2\times 0.06\right)\times 50=56$ Hz.

**Figure 11:**
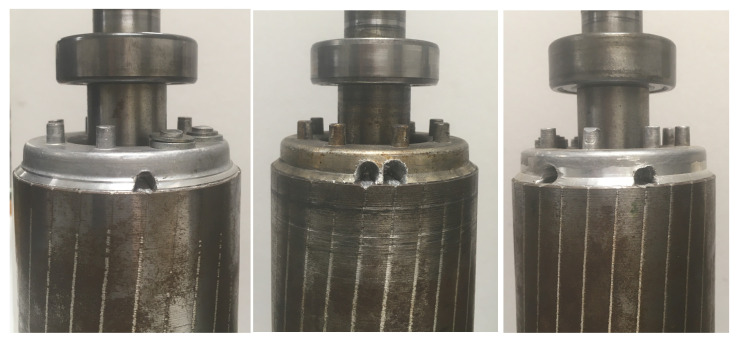
Tested rotors with faulty cages: one broken bar (**left**), two consecutive broken bars (**centre**) and two non-consecutive broken bars (**right**).

In the case of a double bar breakage, the LSH magnitude is a function of the relative position of the two broken bars. In [53], it has been shown that the ratio between the LSH in case of double and single breakages depends on the angle between the broken bars as

(37) $$LSH_{pu}=|\frac{LSH_{double}}{LSH_{single}}|=\left|2\cos\left(p\alpha_{bb}\right)\right|$$

where *p* is the number of pole pairs and $\alpha_{bb}$ is the angle between the two broken bars. From (37), it can be deduced that if $\alpha_{bb}$ approximates π/2p, that is, half a pole pitch, then the second breakage reduces the magnitude of the LSH to a value lower than in the case of a single breakage. Therefore, in this case a motor with two broken bars could be erroneously diagnosed as a healthy motor. This behaviour is more challenging to simulate than the single broken bar fault, and it has been selected to verify the validity of the proposed model for fault diagnosis. Its experimental validation has been carried out using a set of artificially damaged rotors with the three following faults: one broken bar, two consecutive broken bars, and two non-consecutive broken bars, separated half a pole pitch, as seen in Figure 11. An additional healthy rotor, with no broken bars, has been also used for comparison purposes.

**Figure 12:**
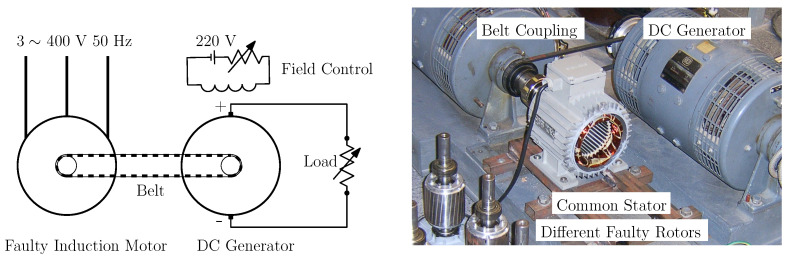
Schema of the loading of the experimental machine (**left**) and experimental setup (**right**). The induction motor under test (Appendix A) is connected to a DC generator via a belt coupling. The DC machine feeds a resistive load. Both the resistive load and the field excitation can be controlled so that the induction machine works at rated speed.

The same stator has been used to perform all the experimental tests, to better control the test conditions in all cases, as seen in Figure 12, right. The induction motor under test (Appendix A) is connected via a belt coupling to a DC generator, which feeds a resistive load, depicted in Figure 12. Both the resistive load and the field excitation of the generator can be controlled, so that the induction machine works at rated speed 1410 r/min (*s* = 0.06). The current of a stator winding has been measured using a sampling frequency of 5000 Hz, during an acquisition time of 50 s.

### 6.2. Analytic Model of the Tested Motor in Healthy Condition

In this section, the characteristic tensors of the tested SCIM (resistance, leakage inductance and main inductance tensors) are obtained for the tested SCIM of Appendix A using the proposed approach.

**Figure 13:**
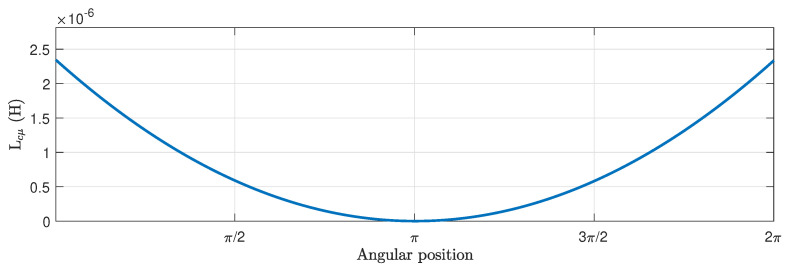
Main inductance between two conductors of the simulated machine, located in the air gap, as a function of their angular separation.

First, the main inductance tensor in the conductors reference frame of Figure 5, $L_{c\mu }$ (13) is built by dividing the air gap circumference of the tested motor into a high number of intervals, *N* = 1008, and applying (14). This value of *N* has been chosen to be a multiple of the number of stator and rotor slots, to avoid numerical errors. The main inductance between two conductors located in the air gap has been represented in Figure 13 as a function of their angular separation, which represents actually the first column of tensor $L_{c\mu }$.

**Figure 14:**
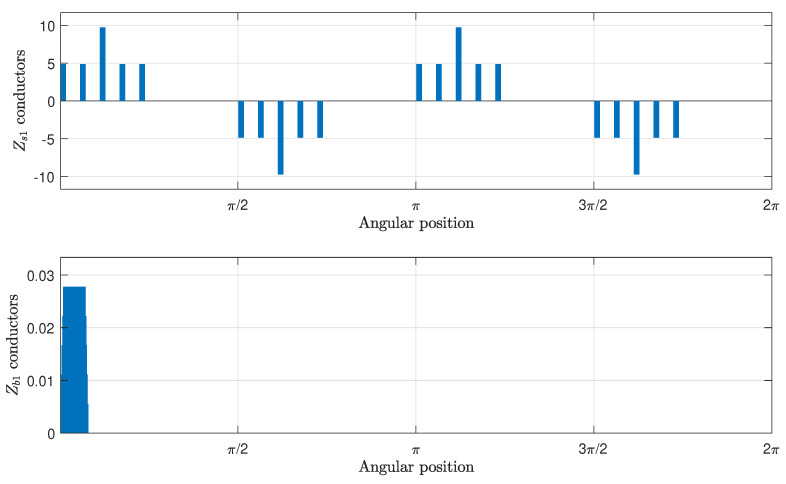
Conductors per air gap interval of a stator winding (**top**) and a rotor bar (**bottom**).

The main inductance tensor, with the mutual inductances between stator windings and rotor bars, is obtained by transforming the tensor $L_{c\mu }$ into the primitive reference frame of Figure 3. This transformation (17) is made using the winding tensor $C_{c}$ (16), which contains the distribution of the conductors of the stator windings and the rotor bars, for each rotor position, as the one shown in Figure 14.

**Figure 15:**
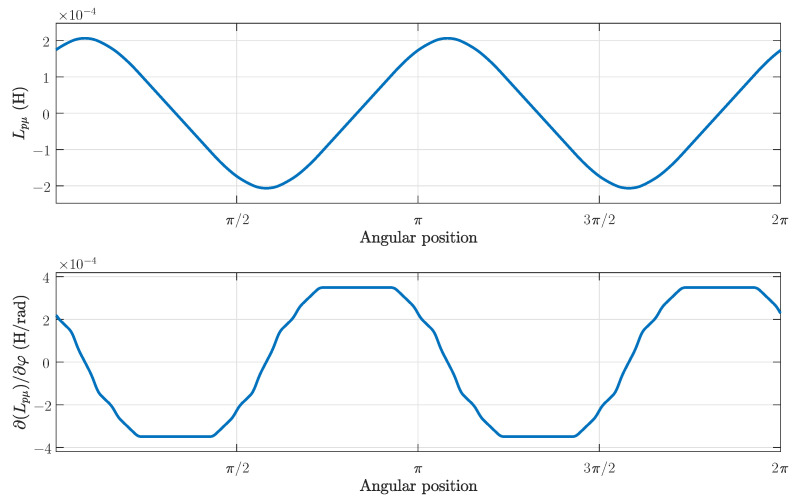
Mutual inductance between a stator winding and rotor bar of the tested machine as a function of the rotor position (**top**), and its angular derivative (**bottom**).

The winding tensor $C_{c}$ has been built using the distributions shown in Figure 14, with the appropriate rotation of their elements for each winding and for each rotor position. The main inductance tensor in the primitive reference frame has been obtained in this step by applying the transformation $C_{c}$ to $L_{c\mu }$ (17). Figure 15, top, presents the main inductance between a stator winding and a rotor bar in the primitive reference frame of Figure 3, and Figure 15, bottom, presents its angular derivative, as a function of the rotor position.

**Figure 16:**
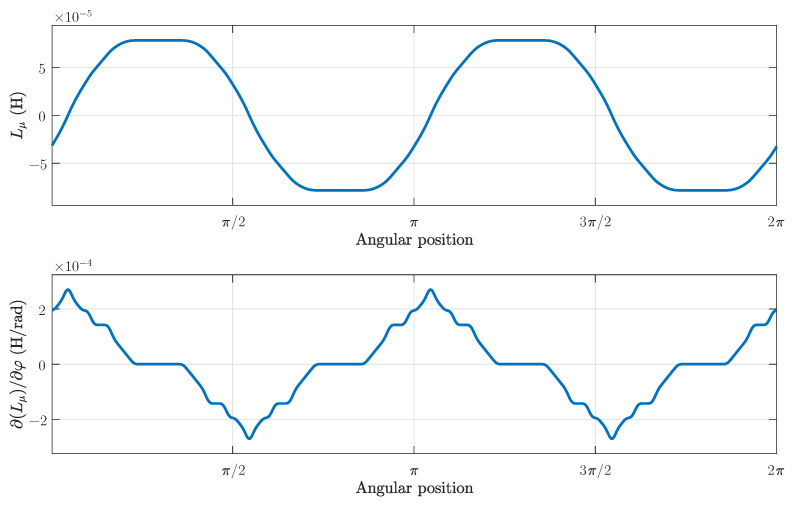
Mutual inductance between a stator winding and a rotor loop of the tested machine as a function of the rotor position (**top**), and its angular derivative (**bottom**).

Applying the connection tensor (25) to the tensors of the primitive reference frame in (21), the machine tensors in the reference frame of Figure 6 are finally obtained for the healthy SCIM. Figure 16, top, presents the main inductance between a stator winding and a rotor loop in the reference frame of Figure 6, and Figure 16, bottom, presents its angular derivative, as a function of the rotor position.

Once obtained the parameters of the tested SCIM in healthy condition, a transformation tensor is used to obtain its parameters under faulty condition. This tensor is different for each type of fault, but it contains only zeros and ones to mark the type and position of the considered cage fault. Its value for the cases of a single broken bar $C_{b_{2}}$ has been given in (27). The tensors for the cases of two consecutive broken bars $C_{b_{2}b_{3}}$ and for the case of two non-consecutive broken bars $C_{b_{2}b_{6}}$ are given by:

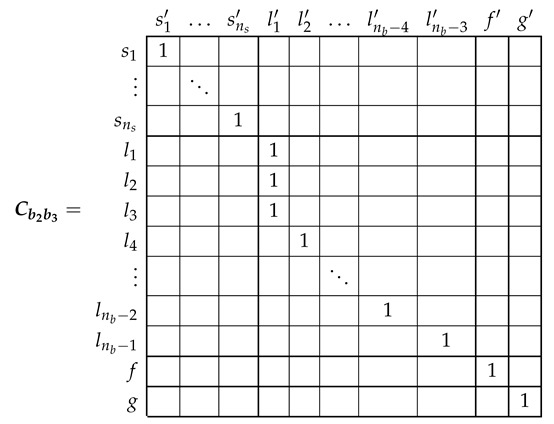
(38)

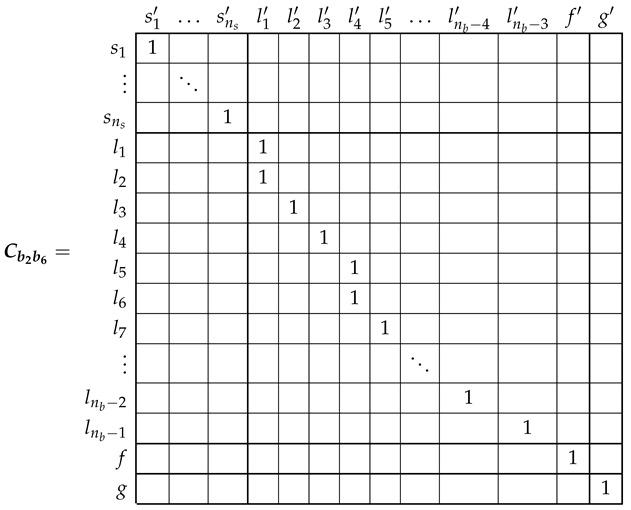
(39)

### 6.3. Comparison between Experimental Tests and Simulations Using the Analytical Model of the SCIM

**Figure 17:**
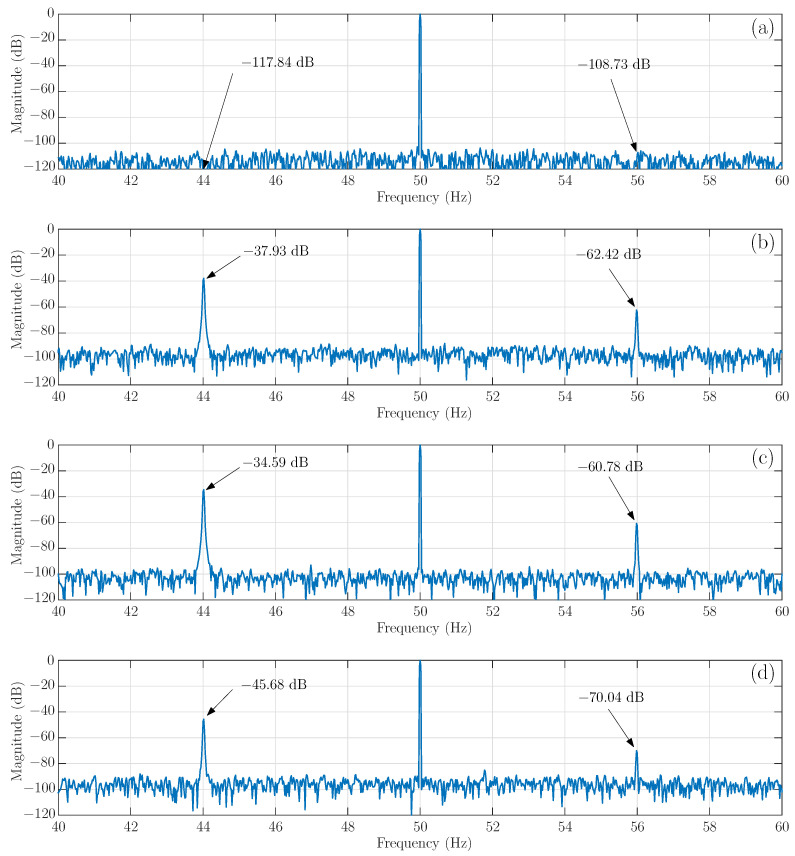
Spectra of the stator current obtained in the simulated tests of the motor of Appendix A with the following cage faults: (**a**) without fault, (**b**) one broken bar, (**c**) two consecutive broken bars (broken bars $b_{2}$, $b_{3}$) and (**d**) two non-consecutive broken bars (broken bars $b_{2}$, $b_{6}$). The main fault harmonics have been marked with text arrows, indicating their magnitude.

The four tested motors have been simulated using the proposed approach, with the model depicted in Figure 2. The spectrum of one of the simulated stator currents is shown in Figure 17, where the main fault harmonics have been marked with text arrows, indicating their magnitude. As predicted by (37), the LSH magnitude increases from the case of a single broken bar, −37.93 dB, Figure 17b, to the case of two consecutive broken bars (broken bars $b_{2}$, $b_{3}$), −34.59 dB, Figure 17c. On the contrary, when the separation between the two broken bars approaches half of a pole pitch (broken bars $b_{2}$, $b_{6}$), the magnitude of the LSH decreases, −45.68 dB, Figure 17d, which may be misdiagnosed as a healthy motor condition.

**Figure 18:**
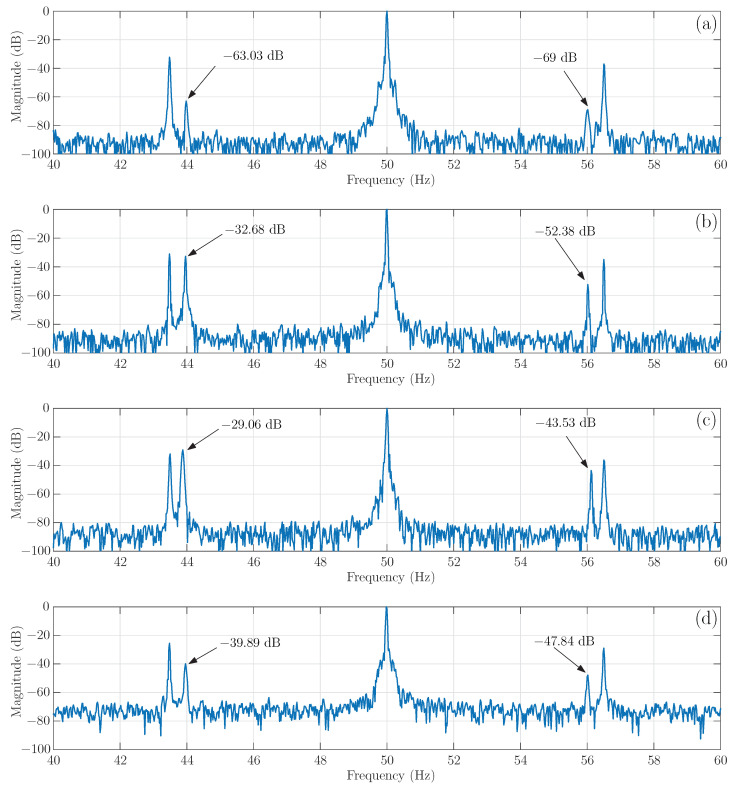
Spectra of the stator current obtained in the experimental tests of the motor of Appendix A with the foll owing cage faults: (**a**) without fault, (**b**) one broken bar, (**c**) two consecutive broken bars (broken bars $b_{2}$, $b_{3}$) and (**d**) two non-consecutive broken bars (broken bars $b_{2}$, $b_{6}$). The main fault harmonics have been marked with text arrows, indicating their magnitude.

The four tested motors have been used for the experimental validation, by recording one of the stator currents with the motor running at rated speed. The spectra of the four experimental currents are shown in Figure 18, where the main fault harmonics have been marked with text arrows, indicating their magnitude. Again, as predicted by (37), the LSH magnitude increases from the case of a single broken bar, −32.68 dB, Figure 18b, to the case of two consecutive broken bars, −29.06 dB, Figure 18c (broken bars $b_{2}$, $b_{6}$). On the contrary, when the separation between the two broken bars is close to a half of a pole pitch, the magnitude of the LSH decreases, −39.89 dB, Figure 18d (broken bars $b_{2}$, $b_{6}$). This situation may be misdiagnosed as a healthy motor condition, especially when the motor has an inherent rotor asymmetry, which generates a small LSH, −63.03 dB, Figure 18a, even in healthy condition. It is worth mentioning that, in the case of the tested motor, two additional harmonics appear at frequencies of 43.5 Hz and 56.5 Hz, due to the belt used for coupling the load to the test bed. In fact, when the motor is tested unloaded, with the belt removed, these harmonic do not appear, so they are probably generated by an axial eccentricity induced by the asymmetric load coupling to the motor shaft. These harmonics do not appear in the case of the simulated motor, because the model presented in this work does not take into account the effect of axial eccentricity.

The results shown in Figure 17 and Figure 18 indicate that the results obtained with proposed model clearly follow the experimental trend. The evolution of the magnitude of the LSH in case of a double bar breakage, compared with the LSH in case of a single broken bar fault, is presented in Table 1, showing a good agreement between simulated and experimental data, which confirms its validity as a tool for fault diagnosis.

The results presented in this section have been obtained also in [53,54], both experimentally and using a bidimensional FEM-based model, which requires greater computing resources than the analytical method proposed in this work. Besides, in [53,54] the broken bar fault was simulated by assigning a very high resistance to the broken bar (10 MΩ), which results in a greater number of unknowns than in the proposed model, and ill-conditioned coefficient matrices. The results of this section can also be obtained with other analytical models presented in the technical literature. In [55], an analytical model is presented, using the winding function approach for obtaining the inductance matrix, which has been extensively used in the technical literature [56]. This approach requires defining many different winding functions between a stator phase and a rotor loop, which depend on their relative positions. Instead, in the proposed method, a simple partial inductance between single conductors has been defined in (14), and the winding tensor (16) provides all the inductances for every relative phase positions using routine tensor algebra (17). The same consideration can be applied to the resistance and leakage inductance matrices, which are obtained in the proposed method using simple tensor transformations of the diagonal matrices (9) and (10), for any type of cage fault. Instead, in the method proposed in [55], the matrices must be built by a careful analysis of the rotor circuit, which are different for each type of fault.

## 7. Conclusions

The application of the tensorial approach proposed by Kron for the analysis of electrical networks to the development of an analytical model of the SCIM with multiple cage faults, as proposed in this work, has proven to be a very effective approach. Starting from a simple, primitive electrical network of the SCIM, which contains the individual stator windings, bars and end ring segments, the complete, complex electrical network of the SCIM with multiple cage faults has been obtained using simple transformation matrices, whose elements are just ones, minus ones and zeros. Besides, this same approach has been applied to obtain the main inductances between stator and rotor windings, starting from a simple primitive framework with air gap conductors, and using a transformation matrix that contains only the conductor distributions of the windings. The proposed method has been theoretically presented and experimentally validated using the diagnosis of single and double breakage faults in the squirrel cage of a commercial induction motor, for consecutive and non-consecutive positions of the broken bars.

The application of this novel approach is not limited to the analysis of rotor cage faults. An added benefit of this approach is that other types of SCIM faults can be added to the model just changing either the primitive networks, as for example in the case of rotor eccentricity, or the transformation matrices, as for example in the case of stator inter-turn short circuits. Future work will include the analysis of simultaneous types of faults, which, in spite of the complexity of the resulting windings configurations, can be addressed in a routine way using the same tensor approach presented in this work. Furthermore, the extension of the proposed approach to other types of electrical machines is currently being addressed.

## Figures and Tables

**Table 1:** Increase of the LSH magnitude in case of a double broken bars fault, at different bar positions, compared with the LSH in case of a single broken bar fault.

	Consecutive Broken Bars	Non-Consecutive Broken Bars
	Broken Bars $b_{2}$, $b_{3}$	Broken Bars $b_{2}$, $b_{6}$
Experimental	3.62 dB	−7.21 dB
Simulated	3.34 dB	−7.75 dB

## Data Availability

Not applicable.

## Appendix A. Commercial IM

Three-phase induction machine. Rated characteristics: P=1.1kW, f=50Hz, U=230/400V, I=2.7/4.6A, n=1410r/min, cosφ=0.8.

Machine dimensions: Effective length of the magnetic core = 70.2 mm, radius at the middle of the air gap = 41.1 mm, air gap length = 1.2 mm.

Stator: Three-phase winding, 36 slots, 78 wires/slot, winding pitch = 7/9, slot opening width = 2.1 mm, phase resistance 7.68 Ω, phase leakage inductance= $2.3\times 10^{-3}$ H.

Rotor: Squirrel-cage winding, 28 bars, slot opening width = 1.4 mm, skew = one slot pitch, rotor bar resistance = $2.02\times 10^{-6}\Omega$, rotor bar leakage inductance = $2.21\times 10^{-7}$ H, end ring leakage inductance = $2.45\times 10^{-8}$ H.

## Appendix B. Current Clamp

Chauvin Arnoux MN60, Nominal measuring scope: 100 mA..20 A, ratio input/output: 1 A/100 mV, intrinsic error: ≤2% + 50 mV, frequency use: 400 Hz to 10 kHz.

## Appendix C. Computer Features

CPU: Intel Core i7-2600K CPU @ 3.40 GHZ RAM memory: 16 GB, Matlab Version: 9.9.0.1592791 (R2020b).
